# Airflow and spectrum interactions jointly regulate gas exchange and biomass production in vertical farming

**DOI:** 10.3389/fpls.2026.1874913

**Published:** 2026-08-26

**Authors:** D. D. Avgoustaki, J. Sohn, A. M. Stamoulou, T. Sachlis, D. Savvidis, G. Matsas, T. Bartzanas

**Affiliations:** 1 Department of Natural Resources and Agricultural Engineering, Agricultural University of Athens, Athens, Greece; 2 Department of Business Development and Technology, Aarhus University, Herning, Denmark

**Keywords:** airflow management, biomass production, controlled-environment agriculture, gas exchange, LED lighting, LED spectra, lettuce

## Abstract

Microclimate heterogeneity and uneven airflow remain major constraints to physiological stability and yield uniformity in vertical farming (VF). While airflow can influence leaf boundary-layer conditions and gas exchange, its interaction with LED spectral composition is not fully understood. This study investigated the combined effects of targeted airflow and LED spectrum on physiological performance and biomass formation of lettuce (*Lactuca sativa* L.) grown in a containerized multilayer VF system. Two lighting treatments were applied: broad-spectrum WHITE and blue–red–far-red (B/R/FR), both at a constant daily light integral (11.5 mol m^-^² d^-^¹; 14 h photoperiod). Targeted airflow was applied at the lower cultivation layer to modify the local canopy microclimate. Airflow significantly increased net photosynthesis under both spectra (p = 0.021), with greater enhancement under WHITE (+24%) than B/R/FR (+11%). Chlorophyll content increased in all airflow treatments (p< 0.001), while stomatal conductance decreased (p< 0.001), resulting in significantly increased intrinsic water-use efficiency (*p*< 0.001). Transpiration declined significantly under B/R/FR with airflow (p = 0.022), which also resulted in reduced leaf area and biomass. These findings demonstrate that airflow enhances net carbon assimilation; however, spectrum-dependent regulation of transpiration and morphogenesis determines biomass translation, highlighting the need for integrated airflow–light optimization in VF systems.

## 1 Introduction

The rapid expansion of controlled environment agriculture (CEA) and, in particular, vertical farming (VF) has positioned vertical farming as a promising solution for sustainable food production in urban and peri-urban areas. By decoupling crop growth from external climatic variability, vertical farms offer precise control over environmental parameters such as temperature, humidity, nutrient supply, and light quality, enabling year-round production with high resource-use efficiency (Avgoustaki and Xydis, 2020; Benke and Tomkins, 2017; Kozai et al., 2019). However, despite these advantages, spatial heterogeneity of the microclimate within vertical cultivation systems remains a critical bottleneck limiting yield uniformity, physiological stability, and economic performance (Touliatos et al., 2016).

Among the environmental parameters shaping plant performance in VFs, airflow distribution plays a pivotal yet often underestimated role. Air movement directly affects leaf boundary layer conductance, influencing transpiration, stomatal behavior, CO_2_ diffusion, leaf temperature, and ultimately photosynthetic efficiency (Jones, 2014). Recent analyses have further shown that even relatively small increases in low air velocity can substantially increase boundary-layer conductance and improve carbon assimilation, although excessive or sustained air movement may also impose hydraulic or mechanical constraints on plant growth (Dupont et al., 2025, 2026). Inadequate or unevenly distributed airflow can lead to localized heat and humidity accumulation within the crop canopy, increasing the risk of physiological disorders such as tipburn in leafy vegetables, particularly in fast-growing species like lettuce (*Lactuca sativa L*.). Previous studies have demonstrated that enhancing airflow can mitigate tipburn incidence by improving calcium transport and reducing leaf surface temperature gradients, while simultaneously affecting gas exchange dynamics (Boulard and Wang, 2000; Bailey et al., 1999; Demers and Gosselin, 2002; Ahmed et al., 2020). Recent lettuce studies have confirmed that the effectiveness of airflow in reducing tipburn depends on its direction, intensity, and interaction with the lighting environment. Downward airflow can improve air penetration toward the inner developing leaves, whereas the combination of light intensity and airflow can modify transpiration, calcium transport, and tipburn severity (Yu et al., 2024; Ertle and Kubota, 2025; Persons et al., 2025).

Lettuce is one of the most widely cultivated crops in vertical farming systems due to its short growth cycle, compact rosette morphology, high nutritional value, and strong sensitivity to microclimatic variations. Its physiological plasticity makes it a suitable model species for investigating plant–environment interactions under controlled conditions. Previous studies have demonstrated that lettuce productivity and quality are highly responsive to photoperiod duration, with optimal performance typically observed under extended light regimes of approximately 14 h per day (Demers and Gosselin, 2002). However, photoperiod effects cannot be considered independently of airflow, as prolonged lighting increases transpiration demand and amplifies the importance of effective convective heat and mass transfer at the leaf surface. Airflow requirements may also change during crop development. As lettuce leaf area and canopy density increase, airflow penetration may decrease, while local aerodynamic resistance and microclimatic gradients within the rosette canopy may become more pronounced. Consequently, constant fan operation does not necessarily produce constant leaf-level airflow throughout the cultivation cycle (Dupont et al., 2025; 2026).

Recent advances in LED technology have enabled highly customizable spectral combinations, allowing growers to tailor light environments to specific developmental stages or production goals. Blue–red–far-red (B/R/FR) spectra have been shown to influence photosynthetic efficiency, leaf expansion, and carbon allocation differently compared to broad-spectrum white lighting (Ouzounis et al., 2015; Zhen and Bugbee, 2020). However, changes in spectral quality may also alter transpiration demand and stomatal regulation, thereby modifying the plant’s sensitivity to airflow and mechanical stress. This interaction suggests that airflow management cannot be optimized independently of lighting strategy, particularly in multilayer vertical systems where vertical gradients in temperature, humidity, and air velocity are common (Taiz et al., 2018). More broadly, vertical farms are increasingly viewed as dynamic production systems in which light, temperature, humidity, CO_2_, and airflow should be adjusted according to plant development rather than maintained as constant set points throughout the production cycle (Kaiser et al., 2024; Heuvelink et al., 2025).

Container-based vertical farms are particularly susceptible to spatial variability in air movement because of their confined geometry, dense crop arrangement and fixed ventilation configuration. Computational fluid dynamics (CFD) studies have demonstrated substantial heterogeneity in air velocity and microclimate conditions within such systems, yet experimental validation linking airflow distribution to plant-level physiological and yield responses remains limited (He and Lee, 2019; Niu and Kozai, 2020). However, most studies investigating airflow in plant factories have primarily focused on microclimate uniformity, tipburn mitigation, or the isolated effects of air velocity on plant growth (Ahmed et al., 2020). Comparatively less attention has been given to whether plant responses to airflow are modified by the spectral environment under which the crop develops. Furthermore, relatively few experimental studies have evaluated the combined effects of airflow and LED spectral composition under the same controlled cultivation conditions, particularly in container-based multilayer vertical farming systems. This knowledge gap is physiologically relevant because airflow and light spectrum regulate several interconnected processes. Increased air movement can reduce leaf boundary layer resistance and modifies convective heat and mass transfer, thereby influencing CO_2_ diffusion, transpiration, and stomatal responses (Jones, 2014; Boulard and Wang, 2000; Bailey et al., 1999). Simultaneously, spectral composition regulates stomatal behaviour, photosynthetic processes, leaf expansion, and biomass allocation through photoreceptor-mediated responses (Ouzounis et al., 2015; Zhen and Bugbee, 2020; Taiz et al., 2018). Consequently, the physiological benefit of enhanced airflow may not translate uniformly into biomass production under different LED spectra. A spectral environment that modifies stomatal regulation, transpiration, or leaf development may alter the capacity of the plant to convert airflow-induced improvements in gas exchange into structural growth. Although these physiological processes are well established in the literature, their combined effects under the experimental conditions used in container-based vertical farming have received comparatively limited experimental investigation.

Despite the individual effects of airflow and LED spectrum being relatively well documented, their combined influence under practical container-based vertical farming conditions remains poorly understood. Most previous studies have investigated airflow primarily in relation to microclimate regulation, tipburn prevention, or plant growth, whereas LED studies have largely focused on spectral optimization independent of airflow management. Consequently, limited experimental evidence is available on how localized airflow modifies lettuce physiological responses under different LED spectral environments, particularly within multilayer cultivation systems where airflow heterogeneity is common. This knowledge gap limits the development of integrated environmental control strategies capable of simultaneously improving crop performance and microclimate uniformity. The present study addresses this gap by experimentally comparing two LED spectral environments under identical cultivation conditions with and without localized canopy-level airflow, thereby providing evidence on their combined influence on lettuce physiology and biomass production.

Therefore, the objective of this study was to evaluate the effects of targeted canopy-level airflow under two LED spectral environments on the physiological performance and productivity of lettuce cultivated in a container-based vertical farming system. Specifically, the study aimed to (i) quantify the effects of localized airflow on photosynthetic rate, stomatal conductance, transpiration, intercellular CO_2_ concentration, and chlorophyll content; (ii) determine whether these physiological responses differed between WHITE and B/R/FR LED lighting; and (iii) assess whether the observed physiological responses were associated with differences in biomass production and morphological development. Rather than investigating airflow or LED spectrum independently, this study experimentally evaluates their interaction under a single controlled cultivation system. The findings contribute experimental evidence on how localized airflow influences lettuce physiological responses under different spectral environments, while recognizing that the conclusions are limited to the airflow conditions, lighting treatments, crop species, and experimental configuration investigated.

## 2 Materials and methods

### 2.1 Plant material and growing conditions

**Figure 1:**
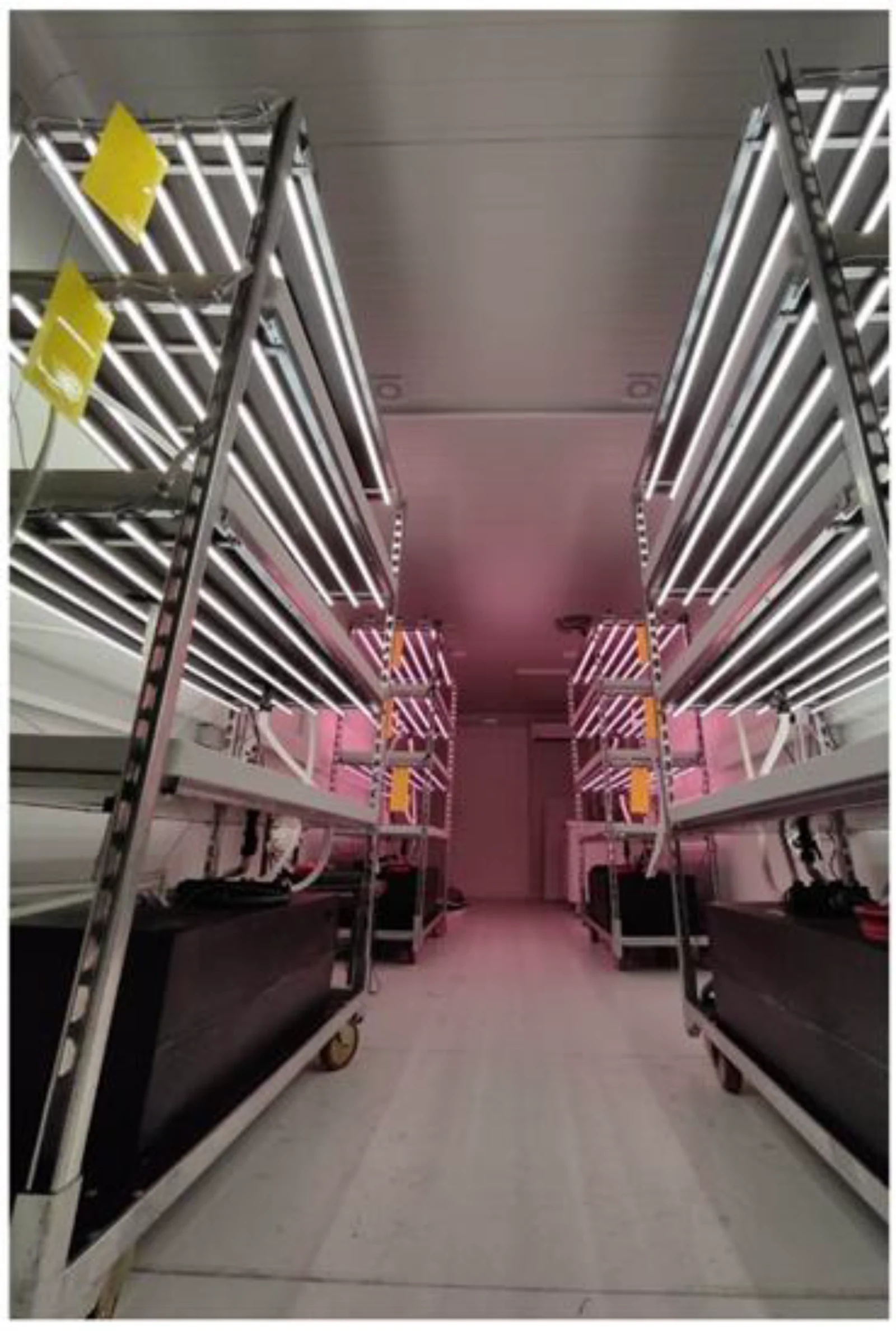
Interior of the vertical farm facility showing the 4 towers with the two different LED spectra (WHITE and B/R/FR).

The experiment was conducted inside the pilot vertical farm of the Laboratory of Farm Structures at the Agricultural University of Athens. The pilot vertical farm was installed inside a 40 ft (≈30 m^2^) container-based cultivation unit with external dimensions of 12 m (L) × 2.44 m (W) × 3 m (H). The farm was equipped with 4 growing towers (T1, T2, T3 and T4) with 3 growing layers (L1, L2, L3) per tower. All growing towers were equipped with a closed-loop Ebb & Flow hydroponic system (**Figure 1**).

**Figure 2:**
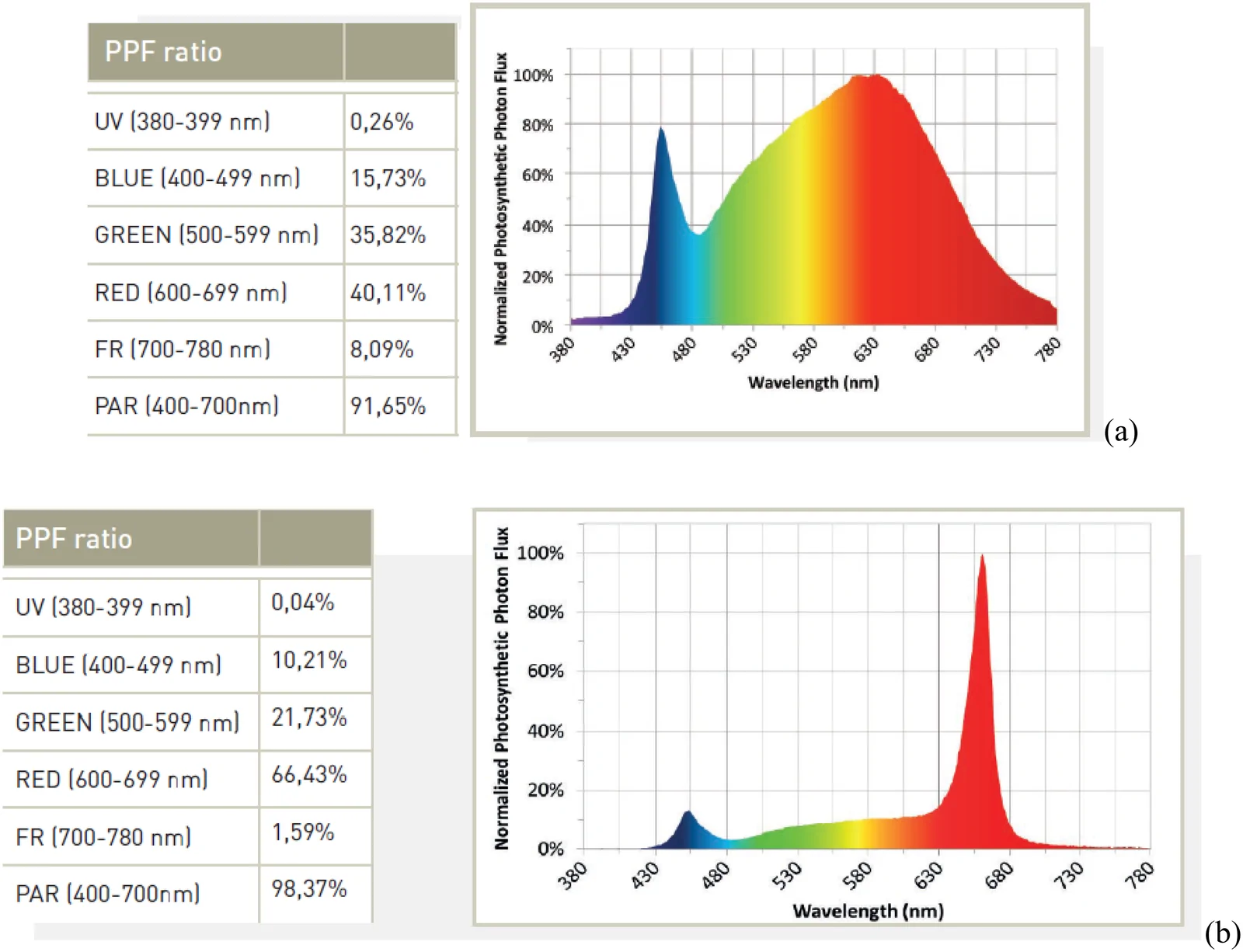
Spectral characteristics of the two LED light treatments. **(A)** White, **(B)** B/R/FR relative spectral photon distribution. X axis: wavelength (in nanometers), Y axis: relative spectral photon distribution %. Quantitative spectral characteristics are summarized in **Tables 2A, B**.

**Figure 3:**
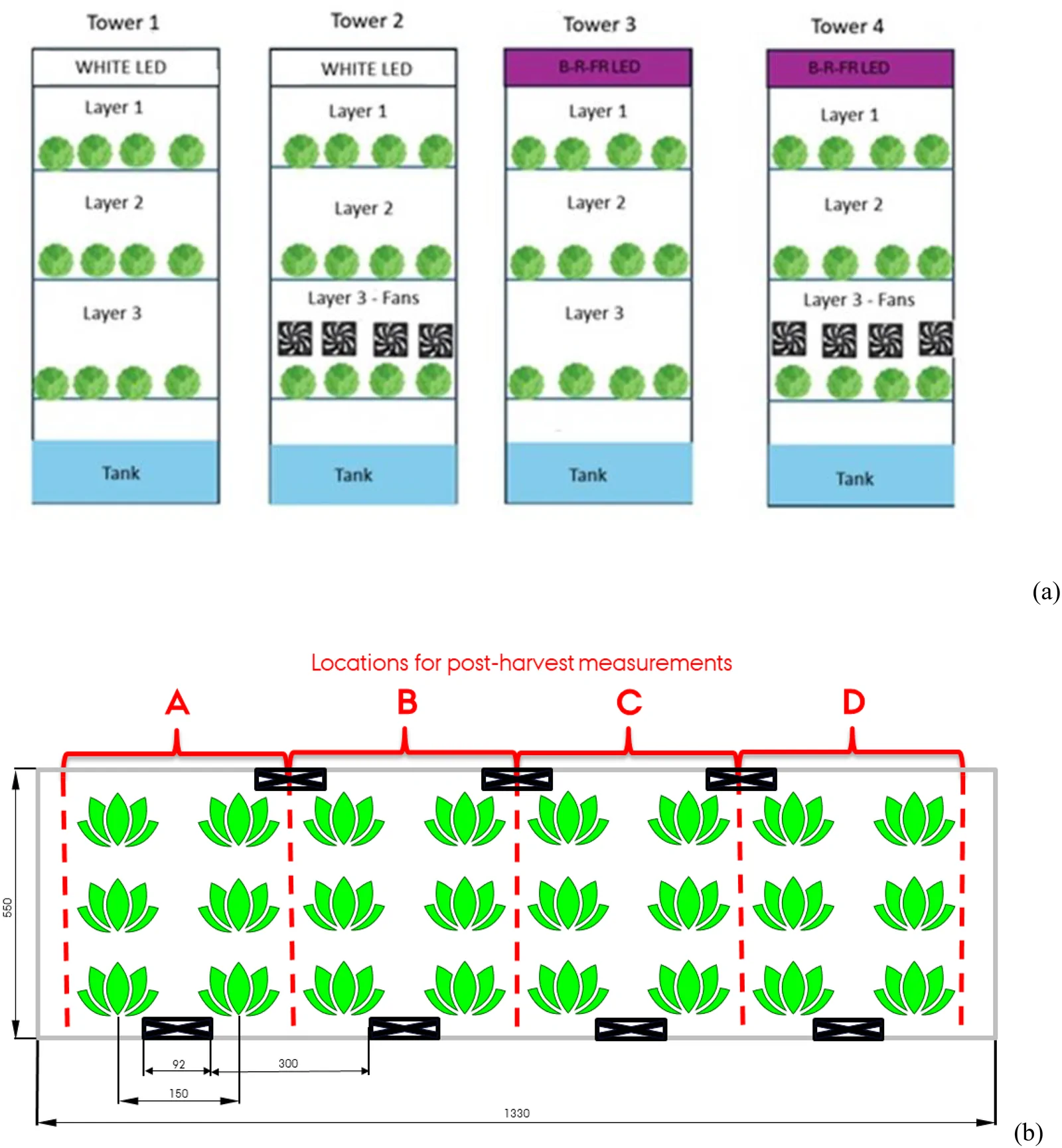
**(A)** Layout of the four towers of the experimental hydroponic system. Towers 1 and 2 are illuminated with WHITE LED lighting, while Towers 3 and 4 are illuminated with B/R/FR LED and **(B)** Airflow direction within the cultivation layer equipped with targeted airflow.

The two towers T1 and T2 were equipped with 18 WHITE LED lamps each (6 in each layer) (**Figure 2B**, **Figure 3A**). Additionally, towers T3 and T4 were equipped with 18 Blue–Red–Far Red (B/R/FR) LED lamps combination (6 in each layer) (**Figure 2A**). **Figure 2** shows the relative spectral output distribution of the different LED treatments. In addition to the spectral distribution shown in **Figure 2**, the principal spectral characteristics of both LED treatments are summarized in the tables of **Figure 2**, including the percentage of emitted photosynthetic photons within the major wavelength bands, including ultraviolet (UV, 380–399 nm), blue (400–499 nm), green (500–599 nm), red (600–699 nm), and far-red (700–780 nm) as also the proportion of photons within the photosynthetically active radiation (PAR, 400–700 nm) waveband. The LED lamps (Vegeled, Colasse, Belgium) at each layer were dimmable to the required light intensity while the photoperiod was automatically controlled using a smart plug system. Each LED lamp had the following dimensions, 141 × 22 cm and power demand of 23–24 W/m. The DLI (Daily Light Integral in mol m^-2^ d^-1^) was maintained at 11.5 mol m^-2^ d^-1^ for all towers and all layers. The corresponding PPFD of the WHITE and the B/R/FR treatments were 250 μmol m^-2^ s^-1^. Spectral measurements were obtained using a calibrated spectroradiometer (model uSPECTRUM, UPRtek) positioned at the canopy level (approximately 40 cm below the luminaires). Measurements were performed at the center of each cultivation layer under steady-state operating conditions. Each tower consisted of three cultivation levels (Levels 1–3) and a recirculating nutrient solution tank located at the base of each tower, supplying the Ebb & Flow hydroponic system through periodic flooding cycles. Targeted airflow was applied at L3 of T2 and T4 using layer-mounted fans (**Figure 3B**).

Lettuce plants (*Lactuca sativa L*.) were selected for this study, as they are leafy vegetable with a small height (rosette formation), short growth cycle high nutritional value, high yield production and high purchase value that makes it a great choice for cultivation inside vertical farms. The planting density in each layer was 10 × 10 cm resulting in 24 plants per layer and 0.76 m^2^ of growing area per layer (31.5 plants/m^2^). In each layer, two-week-old lettuce seedlings were transplanted together with their rockwool growing substrate. The measurements began ten days after transplant, when the plants had developed three pairs of true leaves each. The nutrient solution formulation was obtained from the online platform NUTRISENSE DSS (https://nutrisense.online, accessed on 10 April 2025) (Savvas et al., 2021)) with pH of 5.6 and electrical conductivity (EC) of 2.6 dS/m.

According to previous studies (Demers and Gosselin, 2002), lettuce performs better under an extended photoperiod; therefore, a 14 h of light followed by 10 h of dark cycle was applied daily. In two of the four towers (T 2 and T4, representing one tower from each LED spectral treatment), seven 92 mm fans (ARX CeraDyna Series Axial Fan, 12 V, DC operation, 126 m^3/h, 6W, China) were installed at Level 3 (L3) in order to provide targeted canopy airflow. The fans were installed to be in-between plant columns with around 0.21 m distance between fans as shown in **Figure 3B**. The height of fans was around 15 cm (fan center to surface) above the cultivation bed surface. At both sides (front and back) of the cultivation layer, fans operated as air inlets at around 40% capacity to keep the average air velocity at around 0.4 m s^-1^ within a cultivation bed. The average air velocity was calculated based on measurements collected at a height of 10 cm with distances of 10, 25, and 35 cm in front of each fan. Airflow treatment was applied on day 12 after the transplantation and turned on during the light period (08:00 - 22:00) and turned off during the dark period.

**Figure 4:**
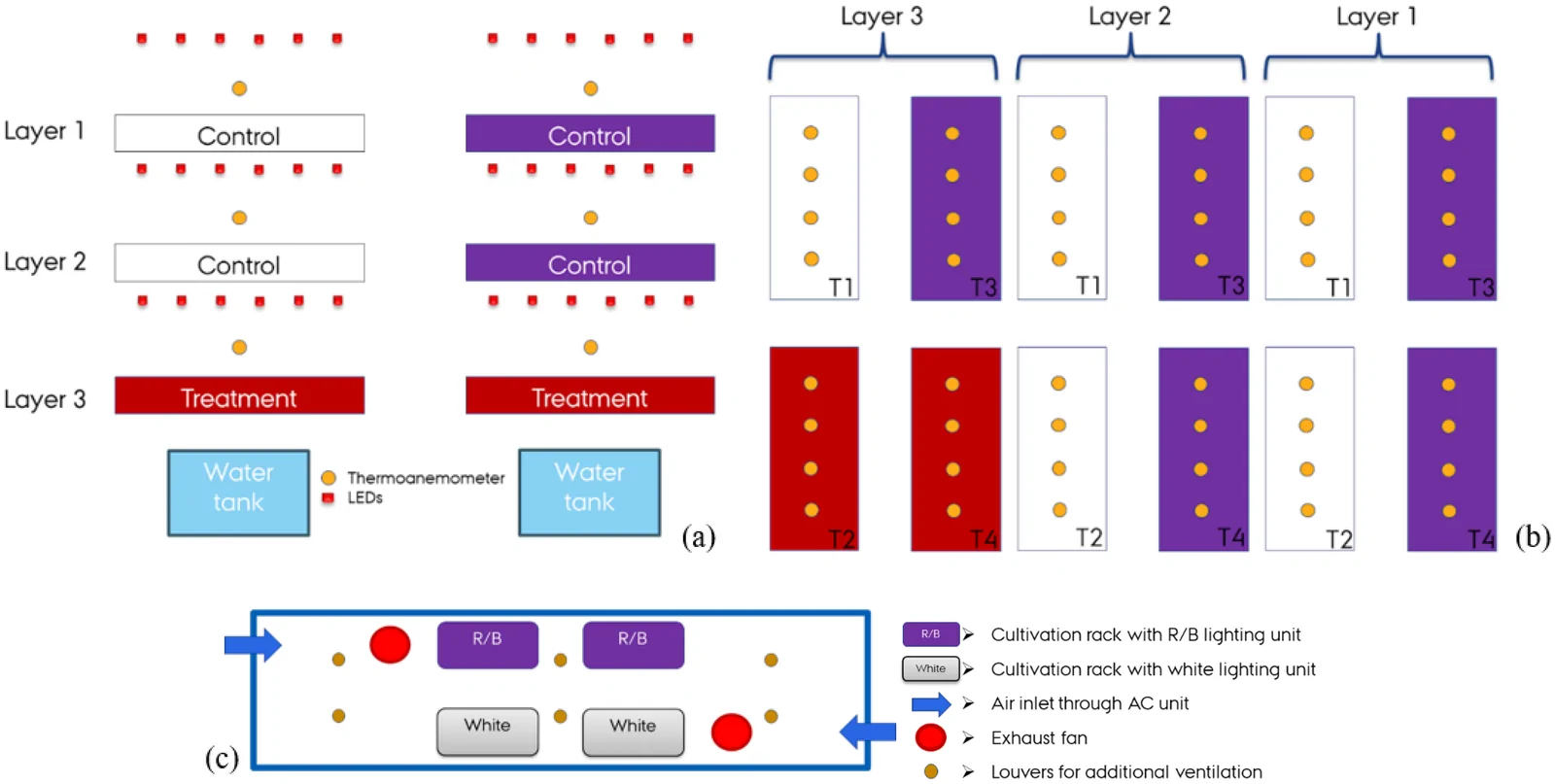
**(A)** Front view graphical representation of the growing towers and the targeted airflow treatments per layer for the two spectral combinations, **(B)** Graphical representation of the towers’ floor plan showing the airflow conditions per layer for the two different spectral combinations and **(C)** Graphical representation of the VF’s floor plan.

The temperature of the space was maintained at 24 °C throughout the experiment using two air conditioning systems (AC Toyotomi, 9000 BTU) on opposite sides of the container, while the relative humidity ranged between 45–60%. The space was ventilated using two exhaust fans (HXM-300, 600CFM, 10.55”) and six 10x10 cm air-inlet grilles installed on the container roof (**Figure 4**).

### 2.2 Data collection

The physiological status of plants was evaluated by daily measurements of the chlorophyll content using the CCM-200 plus sensor (Opti-Sciences, USA). For each growing level, 16 leaf sampling positions were selected and distributed evenly across four zones, with measurements repeated three times daily (10:00, 13:00, and 16:00). The values were expressed in CCI (Chlorophyll Content Index) units and were used to evaluate the physiological status of plants under different airflow conditions. These measurements represented repeated temporal observations within the cultivation layers and were used to characterize the development of chlorophyll responses throughout the experimental period.

Physiological measurements were taken during the advanced stages of plant development, when the plants had developed a full leaf surface. Measurements began 10 days after transplant and continued until the end of the experiment over a 24-day measurement period. Four plants per cultivation layer were selected for gas exchange measurements. The portable gas exchanging sensor, LCi T Portable Photosynthesis System (BioScientific Ltd., ADC, UK) was used to determine various physiological parameters such as photosynthetic efficiency (As in μmol m^-2^ s^-1^), stomatal conductance (gs in mol H_2_O/m^2^s), transpiration rate (E in mmol H_2_O/m^2^s) and Intracellular CO_2_ exchange in the leaf (Ci in μmol CO_2_ mol^-1^).

Intrinsic water-use efficiency (iWUE) was calculated for each paired gas-exchange observation as the ration of net photosynthetic rate to stomatal conductance (iWUE = A/gs) and was expressed as μmol CO_2_ mol^-1^ H_2_O.

At the same time, microclimatic measurements of air temperature (°C), relative humidity (%), and air velocity (m s^-^¹) were taken daily at 12:00 between chlorophyll measurements and were performed using an Air Velocity Meter, Model 9565 SERIES (VelociCalc, TSI Incorporated, USA). The measurements were taken near the surface of the leaves, at four predetermined points on each cultivation layer, to accurately assess the distribution of environmental conditions inside the facility. The four sampling positions were distributed across the cultivation area to characterize spatial variability in the local canopy air environment. Although airflow velocity was measured at multiple positions within the treated cultivation layer to estimate the average canopy air velocity, spatial variability in airflow distribution inevitably remained because of plant architecture, fan placement and canopy development during the cultivation period. Consequently, the reported air velocity represents the average conditions within the treated layer rather than a perfectly uniform airflow field.

Daily measurements were taken using GP1 and GP2 data loggers (Delta-T Devices Ltd., United Kingdom), which provided continuous data storage at 15 minutes intervals throughout the experiment. Temperature and relative humidity sensors were placed at each level of the towers, while pyranometers were installed at selected points to record light flux (LUX), photosynthetic photon flux density (PPFD), and photosynthetically active radiation (PAR). The pH and electrical conductivity (EC) of the nutrient solution were checked daily using a HI9814 GroLine portable instrument (HANNA Instruments) to maintain stable growing conditions.

### 2.3 Plant biomass measurements

The shoot fresh and dry weights were determined after counting the number of leaves and measuring leaf area with the Li-3100 Area Meter (Li-COR Inc., USA). At the end of the experiment, ten plants from each evaluated treatment were harvested for post-harvest measurements. Plant height, number of leaves, shoot fresh weight and leaf area were recorded. In order to collect the dry matter data of the shoots, the shoot samples were placed in a drying oven at 80 °C until constant weight.

Electrical energy consumption associated with the treatments was measured using the PEL103 power-energy logger (Chauvin Arnoux, UK). The electrical energy consumed for the lettuce production was evaluated over the complete crop cycle, considering the electrical demand associated with lighting and targeted airflow operation and the harvested shoot biomass of each treatment. The results were expressed in kWh kg^-1^. Because energy consumption was monitored at the treatment/system level rather than using independently replicated energy-management units, the energy data were interpreted descriptively as a system-level performance indicator and were not used for inferential statistical comparisons.

### 2.4 Statistical analysis

Data were subjected to a two-way analysis of variance (ANOVA). The analyses were performed using SPSS for Windows (IBM SPSS Statistics, version 25.0). The two experimental factors considered were LED spectrum (WHITE and B/R/FR) and airflow condition (no targeted airflow and targeted airflow). The interaction between LED spectrum and airflow was also evaluated. The four environmental combinations included T1L3-C (WHITE × no airflow), T2L3-A (WHITE × airflow), T3L3-C (B/R/FR × no airflow), and T4L3-A (B/R/FR × airflow).

Preliminary comparisons among cultivation layers were conducted to assess baseline differences within each tower. No statistically significant differences were detected among the non-targeted-airflow layers within the corresponding towers. Based on this preliminary assessment and because targeted airflow was specifically applied at Level 3, the main treatment comparison focused on Level 3 of the four towers. The data were tested for homogeneity of variances using Levene’s test.

The experimental design was conducted within a single container-based vertical farming system. Consequently, the airflow treatments should be interpreted as localized environmental manipulations within one experimental facility rather than as independently replicated production units. Statistical analysis therefore evaluates treatment responses under the specific experimental conditions investigated, and extrapolation beyond these conditions should be made with caution.

## 3 Results

### 3.1 Statistical analysis of the physiological traits of lettuce plants

**Figure 5:**
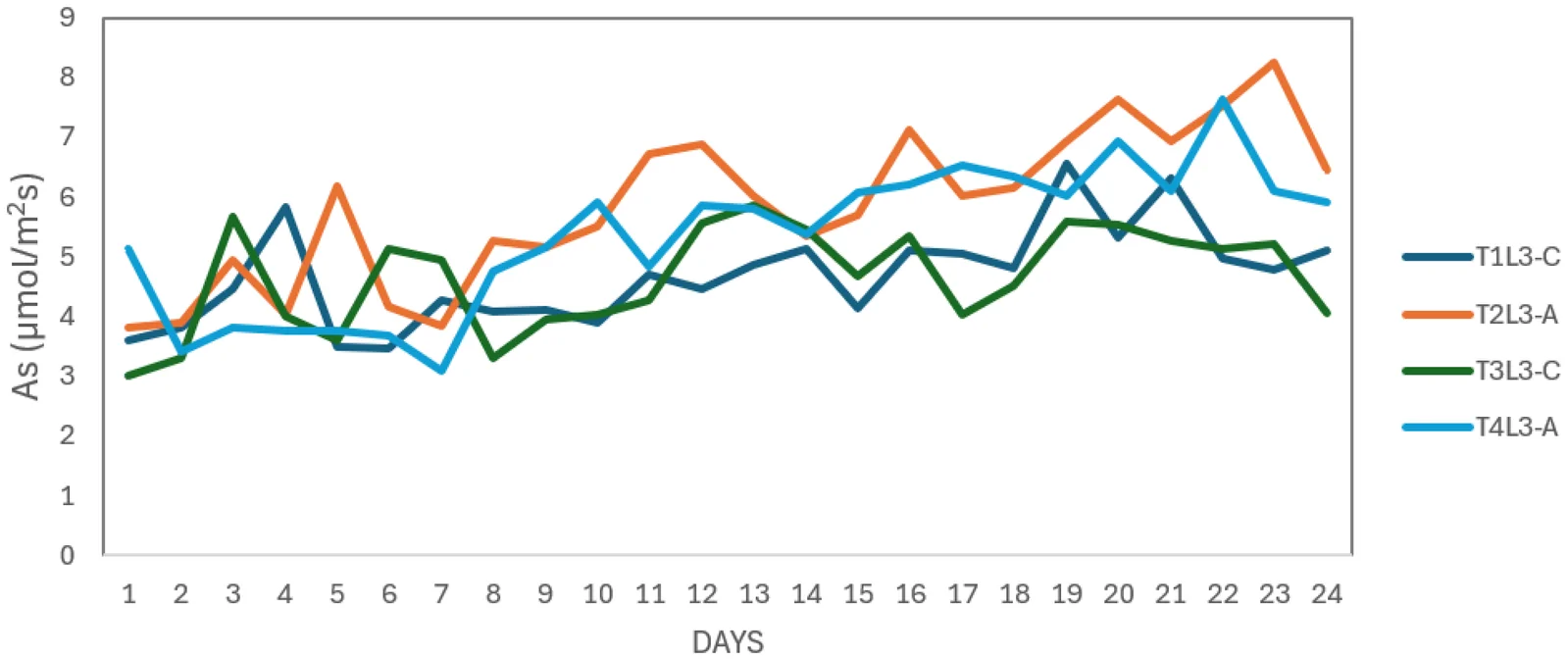
Average daily development of the photosynthetic rate As (in μmol m^-2^ s^-1^), under the four examined treatments of the four treatments T1L3-C (control-WHITE), T2L3-A (airflow-WHITE), T3L3-C (control-B/R/FR) and T4L3-A (airflow-B/R/FR).

To identify statistical differences in terms of photosynthetic rate – As (μmol m^-2^ s^-1^) between the four treatments, the T1L3-C (M = 4.6, SD = 0.8, N = 24) was compared with the T2L3-A treatment (M = 5.7, SD = 1.6, N = 24), the T3L3-C treatment (M = 4.7, SD = 1.2, N = 24) and the T4L3-A treatment (M = 5.2, SD = 1.4, N = 24). Subsequently, two-way ANOVA was used to compare the differences for the photosynthetic efficiency among the four groups and showed that the means between the groups performed statistically significant differences and specifically photosynthetic rate was significantly higher under the targeted airflow treatments, T2L3-A (under the WHITE) and T4L3-A (under the B/R/FR) compared to all the other two control treatments with p= 0.004 (**Table 1**). **Figure 5** illustrates the daily development of photosynthetic efficiency for the four different treatments, showing that the airflow treatments showed higher As compared to the control from DAY 9 of the experiment until the end of the experiment. Spectra combination showed no significant differences between the groups (p=0.376).

**Table 1:** Results of statistical analysis comparing the physiological traits mean values of the four treatments T1L3-C (control-WHITE), T2L3-A (airflow-WHITE), T3L3-C (control-B/R/FR) and T4L3-A (airflow-B/R/FR).

Physiological parameter	TREATMENT	SPECTRUM	AIR	N	MEAN	SD	SOURCE	DF	F	P-value	Partial η²
A (μmol m^-2^ s^-1^)	T1L3-C	W	NoAIR	24	4.6b	0.8	C (AIR)	1	8.582	0.004	0.085
	T2L3-A	W	AIR	24	5.7a	0.6	C (LED)	1	0.792	0.376	0.09
	T3L3-C	BRFR	NoAIR	24	4.7b	0.2	C(AIR):C(LED)	1	0.81	.0370	0.009
	T4L3-A	BRFR	AIR	24	5.2a	0.4	Residual	92			
E (mmol H_2_O/m^2^s)	T1L3-C	W	NoAIR	24	2.2a	0.5	C (AIR)	1	7.493	0.007	0.075
	T2L3-A	W	AIR	24	1.9ab	0.4	C (LED)	1	2.52	0.116	0.027
	T3L3-C	BRFR	NoAIR	24	2ab	0.3	C(AIR):C(LED)	1	0.084	0.772	0.001
	T4L3-A	BRFR	AIR	24	1.7b	0.5	Residual	92			
Gs (mol H_2_O/m^2^s)	T1L3-C	W	NoAIR	24	0.26a	0.1	C (AIR)	1	10.931	0.001	0.106
	T2L3-A	W	AIR	24	0.17b	0.09	C (LED)	1	8.768	0.004	0.087
	T3L3-C	BRFR	NoAIR	24	0.17b	0.08	C(AIR):C(LED)	1	1.829	0.180	0.019
	T4L3-A	BRFR	AIR	24	0.14b	0.08	Residual	92			
CCI	T1L3-C	W	NoAIR	24	25.8b	1.9	C (AIR)	1	33.016	<0.001	0.264
	T2L3-A	W	AIR	24	28.3a	2.4	C (LED)	1	5.375	0.023	0.055
	T3L3-C	BRFR	NoAIR	24	26.8b	1.8	C(AIR):C(LED)	1	0.079	0.779	0.001
	T4L3-A	BRFR	AIR	24	29.5a	2.6	Residual	92			
Ci (μmol CO_2_ mol^-1^)	T1L3-C	W	NoAIR	24	448.5	30.3	C (AIR)	1	4.191	0.043	0.044
	T2L3-A	W	AIR	24	433.5	55.6	C (LED)	1	3.785	0.055	0.04
	T3L3-C	BRFR	NoAIR	24	434.4	35.4	C(TREATMENTS):C(LED)	1	0.159	0.691	0.002
	T4L3-A	BRFR	AIR	24	412.1	52	Residual	92			

The columns represent the sample size (N), mean values (MEAN), standard deviation (SD), Sum of Squares (SS), degrees of freedom (DF), F-test (F), the level of significance (p-value) and Partial η² effect sizes. Significant at the p< 0.05 level. Different lowercase letters indicate significant pairwise differences among treatment combinations.

**Figure 6:**
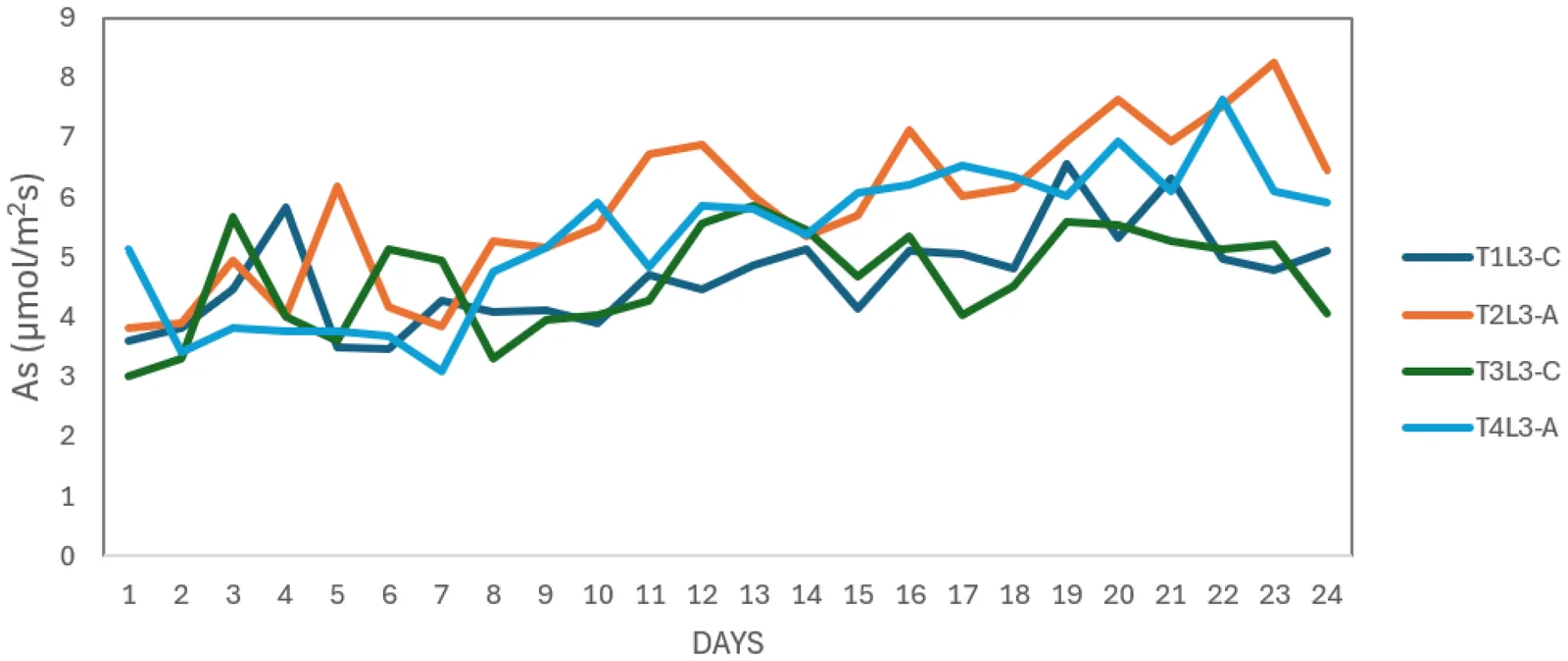
Average daily development of the chlorophyll content (CCI), under the four examined treatments of the four treatments T1L3-C (control-WHITE), T2L3-A (airflow-WHITE), T3L3-C (control-B/R/FR) and T4L3-A (airflow-B/R/FR).

Similar results were retrieved from chlorophyll content (CCI), where the two airflow treatments, T2L3-A and T4L3-A, resulted with statistically significant higher CCI values compared to the two control treatments under both lighting schemes for p-value<0.001 (**Table 1**). Similarly, in **Figure 6** it is observed that the daily development of CCI started increasing compared to the control from day 12 and by the end of the experiment the phenomenon was statistically significant. Furthermore, spectra combination showed no statistically significant difference for the chlorophyll content formation of plants.

**Figure 7:**
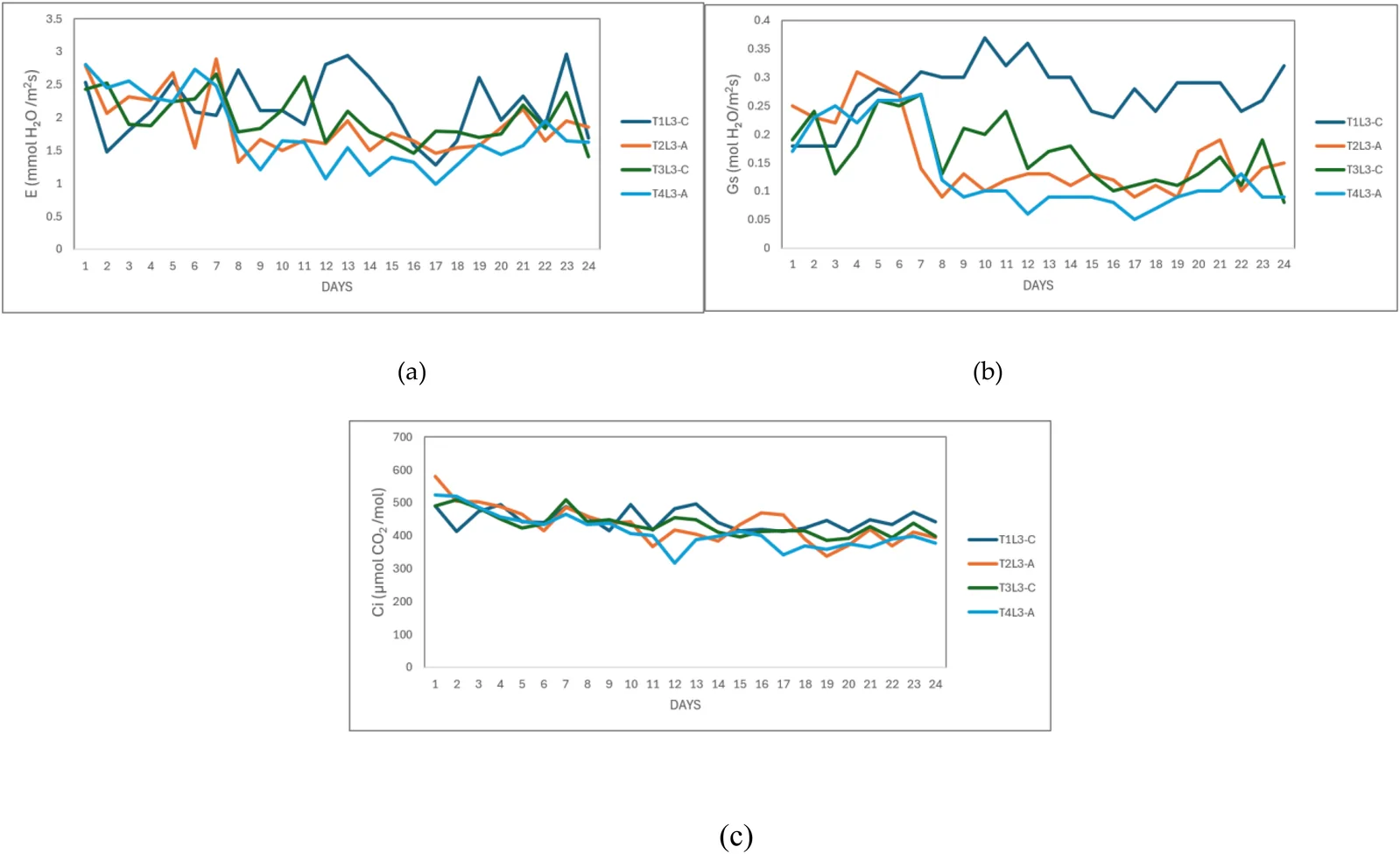
Average daily development of **(a)** transpiration rate (E, mmol H_2_O m^-2^ s^-1^), **(b)** stomatal conductance (Gs, mol H_2_O m^-2^ s^-1^), and **(c)** intracellular CO_2_ concentration (Ci, μmol CO_2_ mol^-1^) under the four examined treatments: T1L3-C (control-WHITE), T2L3-A (airflow-WHITE), T3L3-C (control-B/R/FR), and T4L3-A (airflow-B/R/FR).

Furthermore, data were collected for the transpiration rate – E (mmol H_2_O m^-2^ s^-1^). Specifically, transpiration rate was significantly lower only for treatment T4L3-A under the B/R/FR lighting scheme, compared to the other three treatments (**Table 1**). Spectra combination did not show any significant influence between the groups (p= 0.126) (**Figure 7A**). In terms of stomatal conductance – Gs (mol H_2_O m^-2^s^-1^) the treatment T1L3-C was statistically significant higher compared to the other three treatments with p<0.001. However, spectra combination showed that had significant impact on Gs, where plants that grew under WHITE LED resulted with significantly higher values compared to plants under B/R/FR LED at (1, 92) = 8.768, p= 0.004. **Figure 7B**, shows that stomatal conductance in the WHITE-control treatment was significantly higher compared to the other treatments from the first week of the experiment (day 6). Finally, the intracellular CO_2_ exchange in the leaf – Ci (μmol CO_2_ mol^-1^) showed no statistically significant differences between the treatments under the different light spectra (it is also observed in the daily development of Ci during the experiment at **Figure 7C**), however as it is observed in **Table 1** with treatment T1L3-C performed the highest values compared to the other three treatments. As it is shown in **Table 1** the most important factor that influenced Ci was the air flow with p=0.043.

A two-way ANOVA was conducted to evaluate the effects of LED spectrum and airflow on iWUE. Airflow significantly increased iWUE, F(1, 92) = 39.75, *p*< 0.001, partial η² = 0.302. LED spectrum also had a significant main effect on iWUE, F(1, 92) = 9.51, *p* = 0.003, partial η² = 0.094. However, the interaction between LED spectrum and airflow was not significant, F(1, 92) = 0.18, *p* = 0.675, partial η² = .002, indicating that the effect of airflow on iWUE was similar under both LED spectra. Mean iWUE was [17.5 ± 4] μmol CO_2_ mol^-^¹ H_2_O in T1L3-C, [43.7 ± 9] in T2L3-A, [31.2 ± 12] in T3L3-C, and [54.1 ± 10] in T4L3-A.

### 3.2 Statistical analysis of the post-harvest measurements of lettuce plants

After the finalization of the experiment, 10 plants were harvested from each layer and measurements were conducted to evaluate the quantity variation of lettuce plants under the different airflow conditions between the layers. The yield was measured in terms of the shoot biomass in each of the four treatments. **Table 2** summarizes all the statistical analysis data that was extracted from the post-harvest measurements.

**Table 2:** Results of statistical analysis comparing the quantitative post-harvest mean values of the four treatments T1L3-C (control-WHITE), T2L3-A (airflow-WHITE), T3L3-C (control-B/R/FR) and T4L3-A (airflow-B/R/FR).

	Treatment	Spectrum	Air	N	Mean	SD	Source	DF	F	P-value	Partial η²
Height (cm)	T1L3-C	W	NoAIR	10	25.1	1.3	C (AIR)	1	1.018	0.320	0.028
	T2L3-A	W	AIR	10	25.4	3.2	C (LED)	1	0.222	0.641	0.006
	T3L3-C	BRFR	NoAIR	10	25.8	2.8	C(AIR):C(LED)	1	1.995	0.166	0.053
	T4L3-A	BRFR	AIR	10	24	1.6	Residual	36			
Number of Leave	T1L3-C	W	NoAIR	10	24.8a	2.4	C (AIR)	1	0.741	0.395	0.02
	T2L3-A	W	AIR	10	24.3a	2.2	C (LED)	1	30.856	<0.001	0.462
	T3L3-C	BRFR	NoAIR	10	19.8b	2.1	C(AIR):C(LED)	1	0.115	0.737	0.003
	T4L3-A	BRFR	AIR	10	18.65b	1.5	Residual	36			
Leaf Area (cm^2^)	T1L3-C	W	NoAIR	10	1868a	397	C (AIR)	1	3.108	0.086	0.079
	T2L3-A	W	AIR	10	1928a	378	C (LED)	1	4.274	0.046	0.106
	T3L3-C	BRFR	NoAIR	10	1897a	230	C(AIR):C(LED)	1	5.565	0.024	0.134
	T4L3-A	BRFR	AIR	10	1483b	223	Residual	36			
Fresh Weight (g)	T1L3-C	W	NoAIR	10	96.8ab	7.8	C (AIR)	1	2.483	0.124	0.065
	T2L3-A	W	AIR	10	106a	6.3	C (LED)	1	30.475	<0.001	0.458
	T3L3-C	BRFR	NoAIR	10	84.7b	7.8	C(AIR):C(LED)	1	10.766	0.002	0.23
	T4L3-A	BRFR	AIR	10	58.4c	10	Residual	36			
Dry Weight (g)	T1L3-C	W	NoAIR	10	4.8ab	1	C (AIR)	1	0.932	0.341	0.025
	T2L3-A	W	AIR	10	5.3a	0.9	C (LED)	1	2.777	0.104	0.025
	T3L3-C	BRFR	NoAIR	10	5.1a	0.6	C(AIR):C(LED)	1	8.474	0.006	0.191
	T4L3-A	BRFR	AIR	10	4.1b	0.7	Residual	36			

The columns represent the sample size (N), mean values (MEAN), standard deviation (SD), Sum of Squares (SS), degrees of freedom between the groups (DF), F-test (F), the level of significance (p-value) and Partial η² effect sizes. Significant at the p< 0.05 level. Different lowercase letters indicate significant pairwise differences among treatment combinations.

For the morphological and biomass parameters analysed using TREATMENTS and LED (spectra) as fixed factors, the two-way ANOVA demonstrated significant main effects of both on plant growth traits, including plant height, number of leaves, Leaf Area, Fresh Weight and Dry Weight. Significant interaction effects were also detected for several variables suggesting that plant growth responses to LED spectrum varied according to the applied treatments. Overall, plants exhibited distinct growth and biomass accumulation patterns under different combinations of treatments and lighting conditions. Similarly, significant main effects were identified for most growth and biomass variables. The interaction between spectrum and air flow was significant for selected parameters, indicating that airflow influenced plant response to the lighting conditions applied in each growing layer. These findings suggest that both airflow and LED spectrum conditions play an important role in determining plant morphological development and biomass production.

The leaf area - LA (cm^2^) measured at the end of the experiment obtained with the destructive method showed significant differences after the statistical analysis with the two-way ANOVA. Specifically, treatment under B/R/FR LED and Air, T4L3-A, performed the lowest values of LA compared to all the other three treatments. Spectrum combination had significant effect on Leaf Area (p=0.046) with B/R/FR plants resulting with significantly lower leaf area compared to plants that grew under WHITE LED, while also air flow shows that significantly impacted leaf area (p=0.079), producing better results under the WHITE LED spectrum (**Table 2**).

Similarly, fresh weight – FW (g) and dry weight – DW (g), two-way ANOVA results showed that the same treatment, T4L3-A performed statistically significant lowest values compared to the other three treatments. From **Table 2**, it is observed that air flow did not influence statistically Fresh and dry matter of plants. On the contrary, light spectrum was an important factor as it is observed that plants under WHITE spectra performed significantly higher values compared to plants that grew under B/R/FR lights.

The total biomass of the plants in each treatment was estimated by the following equations:

(1) $$Fresh\;Biomass\;\left(g\;m^{-2}\right)\;=\;Fresh\;Weight\;\left(g\right)/growing\;area\;\left(m^{2}\right)$$

(2) $$Dry\;Biomass\;\left(g\;m^{-2}\right)\;=\;Dry\;Weight\;\left(g\right)/growing\;area\;\left(m^{2}\right)$$

where the growing area was 0.75 m^2^ within each of the harvested plots.

The biomass (calculated with Equations 1, 2) was measured in g m^-2^

**Figure 8:**
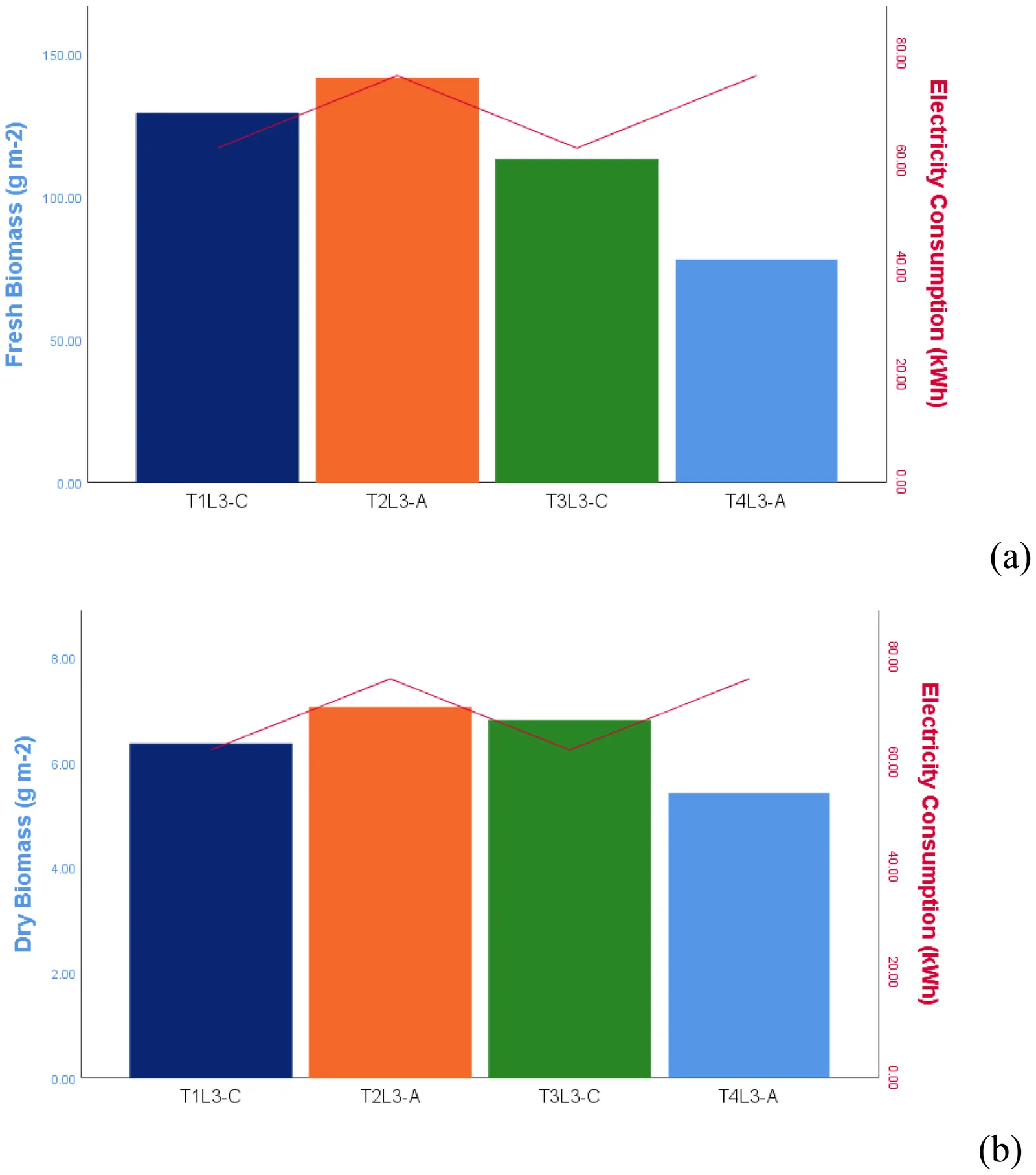
**(A)** The left axis (histograms): The average shoot dry biomass (g m^-2^). The right axis (line): The average electrical energy consumption to produce lettuce (kWh) grown under the four treatments). **(B)** The left axis (histograms): The average shoot dry biomass (g m^-2^). The right axis (line): The average electrical energy consumption to produce lettuce (kWh) grown under the treatments.

**Figure 8** presents the total electrical energy consumption per treatment for the complete crop cycle. The implementation of layer-specific airflow through the operation of side fans resulted in a statistically significant increase in electricity use (+21.6%) in both airflow treatments compared with the respective non-airflow controls. However, the effect of this additional energy input on biomass production was spectrum dependent. A significant increase in fresh and dry biomass was observed only under the WHITE LED treatment, indicating a higher energy-use efficiency of the airflow intervention under this spectral condition. In contrast, the statistically significant lower yield that was detected under the B/R/FR spectrum despite the higher energy demand suggests that the economic and energetic viability of targeted airflow is conditional on the light spectrum, as only the WHITE treatment generated sufficient productivity gains to offset the additional operational energy cost associated with fan use.

## 4 Discussion

### 4.1 Airflow as a regulator of photosynthetic performance and gas exchange

The present study suggests that airflow management significantly affects lettuce physiological performance in container-based vertical farming systems, with the response strongly modulated by LED spectral composition. Airflow application increased photosynthetic rate (As) under both lighting treatments, although the magnitude of the response differed between spectra. Under WHITE LEDs, airflow enhanced As by approximately 24%, whereas under B/R/FR LEDs the increase was approximately 11%, indicating that airflow improved carbon assimilation efficiency regardless of spectral environment, but with greater effectiveness under broad-spectrum lighting.

The observed increase in As may be associated with changes in the leaf microenvironment induced by airflow, potentially including reduced boundary layer resistance and improved CO_2_ exchange at the leaf surface. However, boundary-layer resistance and conductance were not directly measured; therefore, this mechanism should be regarded as a physiologically plausible interpretation rather than an experimentally demonstrated response. In enclosed multilayer cultivation systems, inadequate air circulation frequently leads to localized heat accumulation and reduced convective exchange around the canopy, conditions that can restrict photosynthetic activity despite adequate ambient CO_2_ availability. The absence of significant differences in intercellular CO_2_ concentration (Ci) is consistent with the hypothesis that airflow modified the local gas-exchange environment; however, direct measurements of boundary layer conductance would be required to confirm this mechanism. As lettuce canopy architecture changed throughout the cultivation cycle, the local airflow pattern around the leaves also likely changed despite maintaining a constant fan operation. Consequently, the effective boundary-layer conditions experienced by individual plants probably differed between the early and late stages of crop development. Future studies integrating computational fluid dynamics (CFD) with repeated airflow measurements throughout canopy development would improve understanding of these temporal changes.

Airflow treatments also resulted in significantly higher chlorophyll content index (CCI) values compared with the controls. The higher chlorophyll content observed under airflow treatments indicates improved physiological status, although the underlying physiological mechanisms remain to be further investigated. In the present study, the highest CCI values were recorded under the B/R/FR + airflow treatment; however, this physiological enhancement did not translate into increased biomass production. This finding suggests that elevated chlorophyll concentration and enhanced photosynthetic activity alone are insufficient predictors of productivity in vertical farming systems.

The physiological responses observed suggest that moderate airflow may contribute to improved gas-exchange performance while simultaneously influencing plant development under controlled-environment conditions. Nevertheless, the final agronomic outcome appears to depend on the interaction between airflow-induced physiological responses and spectrum-dependent regulation of plant development.

Although localized airflow modified physiological responses at the treated cultivation layer, the present experiment was not designed to evaluate production uniformity throughout the entire vertical farming system. Therefore, the results should not be interpreted as evidence that the airflow strategy improves crop uniformity at the facility scale. Additional studies including all cultivation layers simultaneously would be required to address whole-system production uniformity.

### 4.2 Spectrum-dependent regulation of photosynthesis and morphology

Under control conditions, photosynthetic rate was nearly identical between WHITE (4.6 μmol m^-^² s^-^¹) and B/R/FR (4.7 μmol m^-^² s^-^¹), indicating that at equal Daily Light Integral (DLI) both spectra supported comparable instantaneous carbon assimilation. However, chlorophyll content was slightly higher under B/R/FR (26.8 vs 25.8 CCI), suggesting enhanced pigment accumulation.

Blue light has been reported to stimulate chlorophyll biosynthesis and regulate stomatal function via cryptochrome-mediated pathways (Hernández and Kubota, 2016; Hogewoning et al., 2010). Additionally, far-red photons influence excitation balance between Photosystem I and II and can enhance photochemical efficiency (Zhen and Bugbee, 2020). The presence of far-red photons in the B/R/FR treatment may therefore have modified the excitation balance between the two photosystems and influenced photochemical performance. Such an effect could contribute to the maintenance of photosynthetic activity under B/R/FR lighting, although chlorophyll fluorescence and photosystem-specific photochemical parameters were not measured in this experiment. Thus, the higher CCI values observed under B/R/FR lighting are consistent with previous reports, although chlorophyll concentration alone cannot explain the observed biomass responses.

Nevertheless, the absence of differences in As between spectra under control conditions suggests that increased pigment concentration did not proportionally enhance net assimilation, likely due to light saturation at the applied PPFD. Because chlorophyll fluorescence, quantum yield and excitation partitioning between Photosystem I and II were not measured, the specific photochemical effects of far-red radiation could not be determined.

Morphologically, however, spectrum-driven differences were evident. Under B/R/FR control conditions, plants exhibited reduced leaf number and lower fresh biomass compared to WHITE control. Red and far-red radiation regulate phytochrome-mediated developmental pathways, influencing leaf initiation, expansion, and carbon allocation (Taiz et al., 2018). Far-red enrichment can modify leaf architecture and biomass partitioning patterns (Zhen and Bugbee, 2020; Meng and Runkle, 2019). Recent lettuce research has also shown that increasing the far-red fraction may promote rapid early growth and morphological responses while increasing the risk of tipburn under some lighting conditions, demonstrating that far-red effects must be interpreted in relation to crop developmental rate and the canopy microclimate (Van de Velde et al., 2025). These observations suggest that differences in spectral quality influenced plant morphology in addition to physiological performance.

### 4.3 Stomatal conductance and iWUE

Stomatal conductance (gs) differed significantly among treatments (p< 0.001), with the WHITE control exhibiting the highest value. Notably, airflow treatments showed higher As despite lower gs, indicating a decoupling between carbon assimilation and stomatal aperture.

This response was further reflected in the calculated intrinsic water-use efficiency (iWUE), which was significantly increased by airflow. Mean iWUE increased from 17.57 to 43.75 μmol CO_2_ mol^-^¹ H_2_O under WHITE lighting and from 31.21 to 54.12 μmol CO_2_ mol^-^¹ H_2_O under B/R/FR lighting. The absence of a significant LED spectrum × airflow interaction (p = 0.675) indicates that airflow consistently improved iWUE under both lighting spectra. These findings suggest that airflow enhanced carbon assimilation per unit stomatal conductance, improving gas-exchange efficiency under controlled-environment conditions.This pattern suggests that airflow altered the relationship between photosynthetic rate and stomatal conductance. Although this response is consistent with improved water-use efficiency, intrinsic water-use efficiency was not directly calculated in this study (Jones, 2014). One possible explanation is that airflow modified the leaf microenvironment, facilitating gas exchange without requiring greater stomatal opening.

Spectral composition also influenced stomatal behavior. Stomatal conductance was lower under B/R/FR control compared to WHITE control, indicating spectrum-dependent regulation. Blue light activates guard cell phototropins, promoting stomatal opening (Shimazaki et al., 2007), whereas red and far-red radiation influence stomatal development via phytochrome signaling (Casson and Hetherington, 2010).

Thus, the lower gs observed under B/R/FR likely reflects altered developmental programming rather than immediate stress-induced closure. The lack of significant differences in Ci further confirms that photosynthesis was maintained despite reduced stomatal aperture.

### 4.4 Transpiration, nutrient transport, and structural growth constraints

Transpiration rate showed a statistically significant reduction only in the B/R/FR + airflow treatment (p = 0.022). Under white lighting, airflow moderately reduced E but did not significantly constrain biomass formation. In contrast, under B/R/FR lighting, airflow reduced transpiration by approximately 15% relative to its control.

Reduced transpiration may influence the transport of water and dissolved nutrients to expanding leaves. Although calcium transport has been associated with transpiration in previous studies, mineral concentrations and nutrient transport were not measured in the present work. Previous studies have shown that reduced transpiration can limit calcium delivery and restrict cell expansion even when photosynthesis remains active (Frantz et al., 2004; Ahmed et al., 2020). The significantly lower transpiration observed in T4L3-A may indicate a reduction in transpiration-driven mass flow of water and dissolved nutrients. However, calcium concentration, mineral composition, nutrient transport and tipburn incidence were not quantified in this study. Therefore, calcium limitation cannot be identified as the cause of the reduced lef area and biomass.

Mechanical stimulation from airflow may also induce thigmomorphogenic responses (plant growth and developmental changes that occur in response to mechanical stimulation, such as airflow), typically reducing leaf expansion and promoting compact growth (Braam, 2005). When combined with far-red-mediated shade signaling, such mechanical cues may generate conflicting growth directives, limiting leaf area development and sink strength.

This interaction explains why T4L3-A exhibited high photosynthetic performance but the lowest leaf area, fresh weight, and dry weight. Carbon assimilation increased without a proportional increase in biomass accumulation, suggesting that additional physiological or developmental processes may have influenced biomass production under B/R/FR lighting.

The absence of significant differences in intercellular CO_2_ concentration among treatments further supports this interpretation, indicating that mesophyll CO_2_ availability was not limiting photosynthesis. Instead, airflow may have altered the physical diffusion environment at the leaf surface, potentially supporting more efficient gas exchange per unit stomatal opening. Direct measurements of boundary-layer conductance would be required to confirm this explanation. This potential physiological adjustment is particularly relevant in VF systems, where water-use efficiency and transpiration-driven nutrient transport must be carefully balanced to avoid disorders such as tipburn. Nevertheless, the present study did not measure tissue nutrient concentration or tipburn incidence, and no direct conclusions regarding calcium transport or tipburn prevention can therefore be drawn.

### 4.5 Yield responses under white and B/R/FR lighting

Airflow improved both physiological efficiency and biomass accumulation under WHITE lighting. Enhanced As translated directly into increased fresh and dry biomass, indicating that carbon assimilation was effectively converted into structural growth.

Conversely, under B/R/FR lighting, airflow enhanced photosynthesis but reduced leaf area and biomass. Previous studies (Zhen and Bugbee, 2020; Taiz et al., 2018) have shown that far-red radiation may influence biomass allocation and plant morphology. The present results are consistent with these observations but do not directly demonstrate the underlying mechanisms. The combination of reduced transpiration, spectral regulation and airflow-associated mechanical stimulation may have contributed to the observed reduction in leaf expansion and biomass; however, the underlying mechanisms were not directly measured.

These findings suggest that airflow optimization cannot be generalized across lighting strategies. The present results indicate that airflow and spectral composition interacted to influence lettuce growth under the experimental conditions used in this study.

### 4.6 System-level implications for vertical farming

The absence of significant differences among control layers indicates acceptable baseline environmental uniformity within the container system. However, the strong responses observed in airflow treatments highlight how localized air velocity modifications can override vertical gradients and reshape physiological behavior. The observed physiological responses are compatible with previous studies (He and Lee, 2019; Niu and Kozai, 2020) reporting spatial airflow heterogeneity in enclosed vertical farming systems.

Although airflow enhanced instantaneous photosynthesis, it did not universally improve energy productivity. Recent evaluations of vertical farming emphasize that productivity should be considered together with energy, land, water, and environmental performance, because maximizing an individual physiological variable does not necessarily maximize whole-system efficiency (Pennisi et al., 2025). Under B/R/FR + airflow, reduced biomass per unit energy input suggests that higher physiological efficiency does not necessarily equate to higher system-level efficiency. These findings provide experimental evidence that may assist the design of airflow management strategies in container-based vertical farming systems. However, further validation under commercial production conditions, multiple crop cycles, and additional crop species is required before broader recommendations can be made (Benke and Tomkins, 2017; Kozai et al., 2019).

The reduced leaf number and leaf area observed under B/R/FR treatments, irrespective of airflow, further indicate a spectrum-driven morphological response. Lettuce grown under red–blue dominant spectra often exhibit more compact architecture and reduced lamina expansion compared to white or broad-spectrum lighting (Ouzounis et al., 2015). While such traits may be advantageous for space efficiency, they can constrain light interception and biomass accumulation if not compensated by higher planting density or optimized airflow distribution. Crop architecture should also be considered when interpreting ventilation responses. Lettuce develops a compact rosette canopy with horizontally expanding and overlapping leaves, which can restrict air penetration toward the inner leaves and promote local humidity accumulation. Recent airflow and CFD studies indicate that airflow direction and canopy structure strongly influence the local microclimate experienced by developing tissues (Persons et al., 2025; Gu and Goto, 2024). Therefore, ventilation strategies established for lettuce may not be directly transferable to crops with upright, open, or vertically elongated canopies.

Importantly, the present results suggest that airflow optimization cannot be generalized across lighting strategies. While airflow improved yield under white LEDs, it imposed a physiological limitation under B/R/FR lighting, likely due to compounded effects on transpiration, morphology, and carbon allocation. This finding addresses a critical gap in VF research, where airflow and lighting are often optimized independently despite their strong physiological interdependence.

Within the experimental conditions investigated, moderate airflow influenced physiological performance and biomass production differently under the two LED spectra, highlighting the importance of considering multiple environmental factors simultaneously. The present study suggestes that moderate airflow can be beneficial, but its interaction with spectral quality must be carefully calibrated to avoid unintended growth penalties.

The experiment focused on a single crop species and growth cycle, which limits extrapolation to longer production periods or fruiting crops. Additionally, while airflow intensity was sufficient to induce physiological responses, finer control of air velocity gradients could further elucidate dose–response relationships. Future studies should integrate real-time physiological monitoring with CFD-informed airflow design and dynamic spectral control to identify optimal combinations that maximize both physiological performance and yield stability.

The present study was conducted using a single lettuce cultivar, one production cycle, one airflow intensity and one container-based VF configuration. Consequently, the findings should be interpreted within the context of these experimental conditions. Future research should include additional crop species, airflow intensities, repeated production cycles, direct measurements of boundary layer conductance and intrinsic water-use efficiency, and nutrient analyses to better elucidate the mechanisms underlying the observed physiological responses.

## 5 Conclusions

This study demonstrated that airflow management and LED spectral composition interact to influence lettuce physiological responses and biomass production in a container-based vertical farming system. Although airflow increased photosynthetic activity and chlorophyll content under both lighting treatments, its effect on biomass production depended on the applied LED spectrum. Airflow significantly increased photosynthetic rate under both WHITE and B/R/FR lighting. However, the greatest improvement in biomass production was observed under the WHITE LEDs, where airflow was associated with higher fresh and dry weights. In contrast, airflow under B/R/FR lighting increased physiological activity but was accompanied by reduced leaf area and lower biomass accumulation. These findings indicate that improvements in physiological performance do not necessarily translate into increased biomass production under all lighting conditions.

Within the experimental conditions investigated, the interaction between airflow and LED spectrum played an important role in determining plant physiological performance and biomass production. Although the observed responses are consistent with changes in leaf boundary-layer conditions and gas-exchange processes reported in previous studies, these mechanisms were not directly measured in the present experiment and therefore remain plausible physiological explanations rather than experimentally confirmed processes. The results suggest that airflow management should be considered together with LED spectral composition when optimizing environmental conditions in VF systems.

Under the conditions examined in this study, WHITE LED lighting combined with targeted airflow resulted in the highest biomass production, whereas B/R/FR lighting combined with airflow enhanced physiological responses without producing comparable biomass gains.

The conclusions of this study are limited to one lettuce cultivar, one production cycle, two LED spectral environments, one airflow intensity, and one container-based vertical farming configuration. Accordingly, the observed physiological and biomass responses should not be generalized to other cultivation systems without additional experimental validation.

Overall, this work provides experimental evidence supporting the need for integrated environmental control approaches in VF systems. Future studies should focus on investigating airflow intensity, dynamic ventilation control, additional crop species and cultivation conditions in order to maximize physiological efficiency, biomass productivity, and overall system sustainability in commercial vertical farming applications.

## Data Availability

The raw data supporting the conclusions of this article will be made available by the authors, without undue reservation.
